# Phase I study of TAS-121, a third-generation epidermal growth factor receptor (EGFR) tyrosine kinase inhibitor, in patients with non-small-cell lung cancer harboring EGFR mutations

**DOI:** 10.1007/s10637-019-00732-4

**Published:** 2019-02-21

**Authors:** Makoto Nishio, Haruyasu Murakami, Yuichiro Ohe, Toyoaki Hida, Hiroshi Sakai, Kazuo Kasahara, Fumio Imamura, Tomohisa Baba, Kaoru Kubota, Yukio Hosomi, Tsuneo Shimokawa, Hidetoshi Hayashi, Kazutaka Miyadera, Tomohide Tamura

**Affiliations:** 1 Department of Thoracic Medical Oncology, The Cancer Institute Hospital, Japanese Foundation for Cancer Research, 3-8-31 Ariake, Koto-ku, Tokyo, 135-8550 Japan; 2 Division of Thoracic Oncology, Shizuoka Cancer Center, 1007 Shimonagakubo, Nagaizumicho, Suntogun, Shizuoka, 411-8777 Japan; 3 Department of Thoracic Oncology, National Cancer Center Hospital, 5-1-1 Tsukiji, Chuo-ku, Tokyo, 104-0045 Japan; 4 Department of Thoracic Oncology, Aichi Cancer Center, 1-1 Kanokoden, Chikusa-ku, Nagoya City, Aichi 464-8681 Japan; 5 Department of Thoracic Oncology, Saitama Cancer Center, 780 Komuro, Inamachi, Kitaadachigun, Saitama, 362-0806 Japan; 6 Department of Respiratory Medicine, Kanazawa University Graduate School of Medicine, 13-1 Takaramachi, Kanazawa City, Ishikawa 920-8641 Japan; 7 Department of Thoracic Oncology, Osaka International Cancer Institute, 3-1-69 Otemae, Chuo-ku, Osaka City, Osaka, 541-8567 Japan; 8 Department of Respiratory Medicine, Kanagawa Cardiovascular and Respiratory Center, 6-16-1 Tomiokahigashi, Kanazawa-ku, Yokohama City, Kanagawa 236-0051 Japan; 9 Department of Pulmonary Medicine and Oncology, Graduate School of Medicine, Nippon Medical School, 1-1-5 Sendagi, Bunkyo-Ku, Tokyo, 113-8603 Japan; 10 Department of Thoracic Oncology and Respiratory Medicine, Tokyo Metropolitan Cancer and Infectious Diseases Center, Komagome Hospital, 3-18-22 Honkomagome, Bunkyo-Ku, Tokyo, 113-8677 Japan; 11 Department of Respiratory Medicine and Medical Oncology, Yokohama Municipal Citizen’s Hospital, 56 Okazawacho, Hodogaya-ku, Yokohama City, Kanagawa 240-8555 Japan; 12 Department of Medical Oncology, Kindai University Faculty of Medicine, 377-2 Onohigashi, Osakasayama City, Osaka 589-8511 Japan; 13 Drug Discovery & Development I, Taiho Pharmaceutical Co., Ltd, 3 Okubo, Tsukuba, Ibaraki, 300-2611 Japan; 14 Thoracic Center, St. Luke’s International Hospital, 9-1 Akashicho, Chuo-ku, Tokyo, 104-0045 Japan

**Keywords:** Epidermal growth factor receptor-tyrosine kinase inhibitor (EGFR-TKI), Non-small-cell lung cancer (NSCLC), Phase I, TAS-121, T790M mutation

## Abstract

*Purpose* We investigated the safety, tolerability, pharmacokinetics, and efficacy of TAS-121, a novel, potent, and highly selective third-generation epidermal growth factor receptor-tyrosine kinase inhibitor (EGFR-TKI) in Japanese patients with advanced EGFR mutation-positive non-small-cell lung cancer (NSCLC) previously treated with EGFR-TKI. *Methods* This was an open-label, non-randomized, multi-center, dose escalation, phase I study conducted in three phases (dose escalation, expansion, and extension phases). TAS-121 was administered orally once daily (QD) or twice daily (BID) under fasting conditions in a 21-day treatment cycle. The primary endpoint was dose-limiting toxicities (DLTs) during Cycle 1 of the dose escalation phase. *Results* In total, 134 patients received treatment. Five and three patients presented a DLT with the QD and BID regimens, respectively. The DLTs were drug-induced liver injury, platelet count decreased, urticaria, interstitial lung disease, and left ventricular failure. The maximum tolerated dose (MTD) was 10 mg/day QD and 8 mg/day BID in the dose escalation phase. The most common adverse drug reactions (ADRs) were dermatological toxicity (89.6%), platelet count decreased (67.2%), and pyrexia (44%) among all patients. Rate of discontinuations due to ADRs at the MTD level were 11.1% with TAS-121 10 mg/day QD and 7.9% with TAS-121 8 mg/day BID. Among 86 T790M-positive patients (confirmed by blood serum sampling in most patients), the objective response rate (ORR) was 28% and highest at 8 mg/day BID (39%). Among 16 T790M-negative patients, the ORR was 19%. *Conclusions* TAS-121 was well tolerated up to the MTD and demonstrated antitumor activity in Japanese T790M-positive NSCLC patients. **Clinical trial registration:** JapicCTI-142651.

**Electronic supplementary material:**

The online version of this article (10.1007/s10637-019-00732-4) contains supplementary material, which is available to authorized users.

## Introduction

The epidermal growth factor receptor (EGFR) is highly expressed in lung cancers [1]. In lung cancer patients, mutations in the EGFR tyrosine kinase (TK) domain are associated with lung tumorigenesis [2] and increased sensitivity to drugs that inhibit EGFR kinase activity [1, 3]. In non-small-cell lung cancer (NSCLC) patients, the frequency of EGFR-TK domain mutations was found to be higher among Asian patients versus patients of other ethnicities (30% versus 8%, *p* < 0.001) [1].

EGFR mutation-positive lung adenocarcinoma patients who were treated with gefitinib, a first-generation EGFR-TK inhibitor (EGFR-TKI), had better outcomes than those without EGFR mutations [4]. Moreover, several EGFR-TKIs, such as gefitinib, erlotinib, and afatinib, were found to be superior to standard chemotherapy (carboplatin-paclitaxel, cisplatin-docetaxel, or platinum-pemetrexed) as an initial treatment for patients with EGFR mutation-positive advanced lung adenocarcinoma/NSCLC in terms of progression-free survival (PFS) and quality of life [4–7]. Therefore, EGFR-TKIs are currently the standard treatment for EGFR mutation-positive NSCLC.

Despite the improvement in clinical outcomes with EGFR-TKI therapy, most patients with EGFR-positive NSCLC develop resistance within 9–14 months [8]. Several mechanisms of resistance to EGFR-TKI therapy have been suggested, including T790M mutation, bypass pathway activation such as through hepatocyte growth factor/mesenchymal-epithelial transition factor (MET) proto-oncogene receptor TK, and small-cell histologic transformation [8, 9]. Among 155 patients with lung adenocarcinoma who presented acquired resistance to erlotinib or gefitinib, the most frequent mechanism of acquired resistance was found to be T790M mutations (63%) [10]. Therefore, there is a clinical need for new EGFR-TKIs that are effective for patients with T790M mutations.

TAS-121 is an orally available, potent, novel third-generation EGFR-TKI that selectively targets EGFR activating and T790M resistance mutations by inhibiting the phosphorylation of mutant forms of the EGFR, including not only the common initial activating mutations (L858R, deletions in exon 19), but also the acquired resistant T790M mutation, while demonstrating moderate to no effect against wild-type EGFR both in vivo and in vitro [11]. This phase I study was the first-in-human study, conducted to investigate the safety and tolerability, pharmacokinetics (PK), and efficacy of TAS-121 in Japanese patients with advanced EGFR mutation-positive NSCLC who had been previously treated with EGFR-TKIs.

Osimertinib, another third-generation irreversible EGFR-TKI, was approved for T790M mutation-positive patients after the present study was conducted. Several mechanisms of resistance to osimertinib have been reported in EGFR T790M-positive NSCLC patients, such as acquired C797S mutation, maintained T790M mutation without acquired C797S mutation, loss of T790M mutation despite the presence of the underlying EGFR activating mutation, loss of EGFR-mutant clones plus alternative pathway activation or histologic transformation, EGFR ligand-dependent activation, and human EGFR-2 and MET amplification [12–14]. There are currently no established treatment options for patients with osimertinib-resistant NSCLC. In the present phase I study, efficacy after osimertinib treatment was assessed in an exploratory manner.

## Methods

### Study design and treatment

This was an open-label, non-randomized, dose escalation phase I study conducted between December 14, 2015 and July 7, 2016 at 12 centers in Japan. The study was conducted in three phases (dose escalation phase, expansion phase, and extension phase), and the design is shown in Supplementary Fig. 1 (Online Resource 1). In each phase, TAS-121 was administered orally once daily (QD) or twice daily (BID) under fasting conditions to all patients in a 21-day treatment cycle. In patients who received the BID regimen, the administration interval was ≥10 h.

The first phase was the dose escalation phase conducted according to a 3 + 3 design, with a minimum of three patients treated at each dose level. In this phase, TAS-121 was administered orally QD with a starting dosage of 2 mg/day. The dosing rationale was derived from a 4-weeks repeated oral-dose toxicology study in monkeys. In the preclinical study, the highest non-severely toxic dose was 2 mg/kg/day, which was converted to 6.48 mg/body/day for the human equivalent dose. Considering the cardiovascular toxicity observed in the safety pharmacology study, the starting dosage for this first-in-human study was determined to be 2 mg/body/day. At Dose Level 1 (starting dose) in this dose escalation phase, at least three patients received 2 mg/day of TAS-121, and the dosage was increased from 2 mg/day (on Day −2) to 4 mg/day (on Day 1), 2 days after the first administration. If none of the initial three patients treated within a dose level experienced a dose-limiting toxicity (DLT) during Cycle 1, the dose was escalated to the next level. If a DLT was observed in one of the first three patients, then three additional patients were enrolled at the same dose level. If none of the three additional patients experienced DLTs after all of them completed Cycle 1, the dose was escalated to the next level. If at least one of the additional three patients developed a DLT, then the dose escalation was stopped and at least six evaluable patients were enrolled at the previous dose level to establish the maximum tolerated dose (MTD). The maximum dose level was defined as Dose Level 9 (150 mg/day). Based on safety and PK results, the dosing schedule could be changed to a BID regimen. The MTD was defined as the highest dose level at which <33% of the patients experienced a DLT during Cycle 1.

The expansion phase was conducted in two parts (first and second stage). Patients were allowed to enter the expansion phase if either of the following criteria were met: if they showed complete response (CR) or partial response (PR) based on Investigator assessment, or if ≥33% patients experienced a drug-related ≥Grade 2 diarrhea or ≥Grade 1 rash in one dose level. In the first stage of the expansion phase, the maximum starting dose level of TAS-121 was defined as one dose lower than the dose level in which DLTs at the dose escalation phase were evaluated. In the second stage of the expansion phase, the maximum starting dose level was the MTD. In the expansion phase, DLTs were not assessed.

The extension phase was conducted in four cohorts (A, B, C, and D) to investigate the safety, PK, and antitumor activity observed with the MTD or lower dose determined in the dose escalation phase. Cohort A comprised patients with T790M mutation-positive NSCLC (confirmed by blood serum sampling) and who had received prior EGFR-TKI therapy as first-line treatment. Cohort B comprised and compared T790M mutation-positive and T790M mutation-negative NSCLC patients who had received at least two prior therapies, with the immediate prior treatment being gefitinib, erlotinib, or afatinib before allocation. Cohort C comprised patients with progressive disease (PD) after osimertinib therapy for NSCLC. Cohort D comprised NSCLC patients with G719X activating mutation in the EGFR.

The main discontinuation criteria were as follows: upon patient request; lack of efficacy of treatment; unacceptable adverse events (AEs); dose interruption >21 days; need for >2 dose reductions or reduction from 2 mg/day of the study drug; physician’s discretion; or pregnancy. Patients were not allowed to receive any other investigational treatment, or any other anticancer treatment, including chemotherapy, immunotherapy, biological response modifiers, or anti-neoplastic endocrine treatment. Palliative radiotherapy was not permitted while the patient received the study treatment.

The study was conducted in accordance with Good Clinical Practice and International Council for Harmonisation of Technical Requirements for Pharmaceuticals for Human Use guidelines, and the ethical principles laid out in the Declaration of Helsinki. The study protocol was approved by the institutional review board of each participating center. All patients provided written, informed consent to participate. This study was registered at JapicCTI (No. JapicCTI-142651).

### Patients

The inclusion criteria were male and female patients ≥20 years of age with histologically or cytologically confirmed NSCLC; Eastern Cooperative Oncology Group performance status of 0 or 1; and able to take oral medication. Patients had to have documented evidence of any activating mutation in the EGFR, and prior treatment with EGFR-TKIs. The main inclusion criteria for specific study phases/stages were as follows: for the dose escalation phase and first stage of the expansion phase, no standard treatment; for the second stage of the expansion phase, T790M mutation in the EGFR as determined by polymerase chain reaction-based testing of either a blood or tumor sample; and for the expansion phase (Cohort C), immediate prior treatment with osimertinib before allocation and radiological documentation of disease progression following the osimertinib treatment.

The main exclusion criteria were as follows: prior treatment with an EGFR-T790M inhibitor (applies to the second stage of the expansion phase and Cohorts A, B, and D of the extension phase); evidence of corneal disorder/keratopathy, cardiac arrhythmia or conduction abnormality; vomiting within 24 h prior to the day on which the study drug was scheduled to be administered; unresolved toxicity of ≥Grade 1 attributed to any prior therapies (excluding alopecia and skin pigmentation); serious illness or medical condition; or uncontrollable pleural effusion. A complete list of the inclusion and exclusion criteria is provided in the Supplementary Methods (Online Resource 2).

### Endpoints

The primary endpoint was DLTs, with only DLTs during Cycle 1 of the dose escalation phase considered in the assessment. Secondary endpoints were the objective response rate (ORR), disease control rate (DCR), and PFS as efficacy, along with the PK profile of TAS-121 and any preliminary antitumor activity observed with TAS-121.

### Assessments

#### Safety and tolerability

AEs were evaluated and graded according to the National Cancer Institute Common Terminology Criteria for Adverse Events Version 4.03. DLTs were also evaluated and defined as hematologic toxicity (Grade 4 neutropenia lasting >7 days, Grade 4 thrombocytopenia or Grade 3 thrombocytopenia associated with bleeding and requiring blood transfusion, or febrile neutropenia) or non-hematologic toxicity (Grade ≥3 nausea/vomiting uncontrolled by aggressive antiemetic treatment, Grade ≥3 diarrhea lasting >48 h and unresponsive to treatment, Grade ≥3 aspartate aminotransferase/alanine aminotransferase lasting >7 days, corneal disorder worsening by ≥1 grade, or Grade ≥3 other non-hematological toxicity).

#### Pharmacokinetics

TAS-121 PK parameters included the terminal phase elimination half-life (T_1/2_), time to maximum plasma concentration (T_max_), maximum plasma concentration (C_max_), area under the plasma concentration-time curve from time 0 to 24 h (AUC_0–24 h_) for the QD dosing regimen and area under the plasma concentration-time curve from time 0 to 12 h (AUC_0–12 h_) for the BID dosing regimen. PK parameters were evaluated on Day 1 and Day 15. Concentrations of TAS-121 were measured in plasma by validated bioanalytical methods using liquid chromatography-tandem mass spectrometry.

#### Pharmacogenomics

To assess the EGFR T790M mutation tumor status, mandatorily collected plasma from patients was processed, and isolated circulating cell-free DNA (cfDNA) was submitted for laboratory testing (Fluorescence Resonance Energy Transfer-based Preferential Formation Assay [F-PHFA] method) by central review. In some patients, collected tumor biopsies were submitted for EGFR genotyping (Therascreen® EGFR RGQ PCR kit [Qiagen, Venlo, Netherlands]).

#### Efficacy

Antitumor efficacy was based on objective tumor assessments according to the Response Evaluation Criteria in Solid Tumors criteria (version 1.1, 2009). Computed tomography scans were performed at baseline, every 6 weeks after starting administration, and at the time of discontinuation. The ORR, DCR, and PFS at each phase were assessed according to dose level and regimen. ORR was defined as the proportion of patients in which the best overall response was determined to be CR or PR and was calculated in patients with measurable lesions. DCR was defined as the proportion of patients in which the best overall response was determined to be CR, PR, or stable disease. PFS was defined as the median time from enrollment to PD or death from any cause**.**

### Statistical analysis

The planned sample size was 300 patients maximum, including 54 patients in the dose escalation phase, seven patients at each level of the first stage of the expansion phase, 20 patients at each level in the second stage of the expansion phase, and in the extension phase, 100, 40, 20, and 10 patients in Cohorts A, B, C, and D, respectively. The main analysis sets in each study phase are defined in Supplementary Table 1 (Online Resource 3). Safety and efficacy data are summarized using descriptive statistics. PK parameters were calculated according to the non-compartmental method. ORR, DCR, and median PFS were calculated along with 95% CIs. The statistical software used to perform statistical analyses was SAS version 9.2 (SAS Inc., Cary, NC, USA, RRID: SCR_008567). For the PK analysis, Phoenix® WinNonlin® Ver. 6.3 and 6.4 (Certara, Princeton, NJ, USA) was used.

## Results

### Patients

**Fig. 1:**
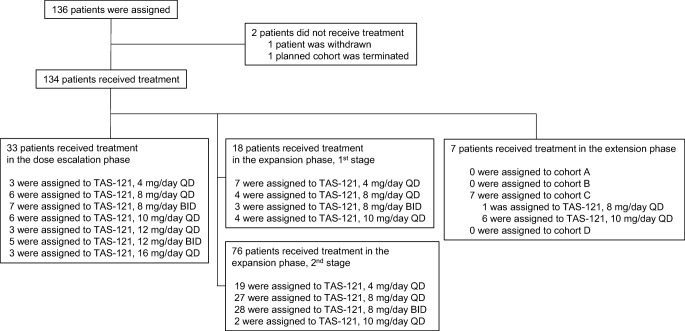
**Patient disposition**. *Abbreviations: BID* twice daily; *QD* once daily

The patient disposition is shown in Fig. 1. A total of 134 patients received treatment, among whom 33 were enrolled in the dose escalation phase, 94 were enrolled in the expansion phase (first stage, 18 patients; second stage, 76 patients), and seven were enrolled in the extension phase. There was no assignment to Cohorts A, B, and D of the extension phase, and only Cohort C (seven patients) was opened.

Patients’ background characteristics in the dose escalation/first stage of the expansion phase, the second stage of the expansion phase, and the extension phase (Cohort C) are shown in Table 1. Most patients were female (57.1%–77.6%), and the median age ranged between 64 and 66 years. The most common histologic type was adenocarcinoma. The median number of prior treatments in all groups was three, and that of prior EGFR-TKI treatments was one in the dose escalation/first stage of the expansion phase and in the second stage of the expansion phase, and two in the extension phase (Cohort C). In most patients in each group, the last treatment received before the present study was EGFR-TKI treatment. Regarding EGFR mutation type by local test, the most common mutation type among the study patients was exon 19 Del, followed by L858R. Regarding T790M status by central test, 56.9% (29/51) of patients in the dose escalation/first stage of the expansion phase and 100% (76/76) of patients in the second stage of the expansion phase were diagnosed as EGFR T790M-positive in cfDNA analysis using F-PHFA or the Therascreen® test.

**Table 1:** Patient background characteristics

Characteristic	Dose Escalation/Expansion 1st stage		Expansion 2nd stage		Extension Cohort C	
	*N* = 51		*N* = 76		*N* = 7	
Sex, n (%)						
Male	21	(41.2)	17	(22.4)	3	(42.9)
Female	30	(58.8)	59	(77.6)	4	(57.1)
Age, years						
Median (Min, Max)	64	(39, 80)	65	(35, 82)	66	(53, 70)
Performance status, n (%)						
0	18	(35.3)	26	(34.2)	3	(42.9)
1	33	(64.7)	50	(65.8)	4	(57.1)
Histological type, n (%)						
Adenocarcinoma	51	(100)	74	(97.4)	7	(100)
Squamous-cell carcinoma	0	(0)	2	(2.6)	0	(0)
No. of prior treatments						
Median (Min, Max)	3	(1, 16)	3	(1, 21)	3	(2, 5)
No. of prior EGFR-TKI						
Median (Min, Max)	1	(1, 6)	1	(1, 7)	2	(2, 3)
Last treatment before study start, n (%)						
EGFR-TKI	33	(64.7)	49	(64.5)	7	(100)
Other treatments	18	(35.3)	27	(35.5)	0	(0)
EGFR mutation type by local test, n (%)						
Exon 19 Del	33	(64.7)	45	(59.2)	5	(71.4)
L858R	18	(35.3)	31	(40.8)	2	(28.6)
Other	1	(2)	0	(0)	0	(0)
T790M status by central test, n (%)					No data	
Positive	29	(56.9)	76	(100)		
Negative	22	(43.1)	0	(0)		

*EGFR-TKI* epidermal growth factor receptor-tyrosine kinase inhibitor

### Safety and tolerability

Safety results of each dose level were collected and analyzed by the sum of patients in all phases (escalation, expansion, and extension phases). The DLTs are shown in Table 2. The numbers of patients who presented a DLT with the QD regimen was one patient who received 10 mg/day (drug-induced liver injury), two patients who received 12 mg/day (platelet count decreased and urticaria), and two patients who received 16 mg/day (urticaria and interstitial lung disease). With the BID regimen, one patient who received 8 mg/day presented a DLT of interstitial lung disease; among two patients who received 12 mg/day, one patient presented a DLT of interstitial lung disease, and another patient presented two DLTs (platelet count decreased and left ventricular failure). The MTD was determined to be 10 mg/day QD and 8 mg/day BID in the dose escalation phase. In the dose escalation phase DLT assessment of the 4 mg/day, 8 mg/day, and 16 mg/day QD dosages commenced in order of dose. Furthermore, DLT assessment of the 10 mg/day QD and 12 mg/day QD dosages commenced additionally after the assessment of the 16 mg/day QD dosage.

**Table 2:** Dose-limiting toxicity

Regimen	Dose	Number of DLT evaluable patients	Number of patients who experienced a DLT	DLT	Grade
QD	4 mg/day	3	0	–	–
	8 mg/day	6	0	–	–
	10 mg/day	6	1	Drug-induced liver injury	G3
	12 mg/day	3	2	Platelet count decreased	G4
				Urticaria	G3
	16 mg/day	3	2	Urticaria	G3
				Interstitial lung disease^a^	G3
BID	8 mg/day	6	1	Interstitial lung disease^a^	G3
	12 mg/day	5	2	Interstitial lung disease^a^	G3
				Platelet count decreased	G3
				Left ventricular failure	G3

*BID* twice daily, *DLT* dose-limiting toxicity, *QD* once daily

^a^Interstitial lung disease included lung disorder and pneumonitis

Adverse drug reactions (ADRs) with an incidence of ≥10% by dose are shown in Table 3. The most common ADRs of any grade were dermatological toxicity (89.6%, 120/134), platelet count decreased (67.2%, 90/134), and pyrexia (44.0%, 59/134) among all patients. The incidence of interstitial lung disease was 7.5% (10/134) and all events were manageable. The incidence of embolic and thrombotic events was 17.9% (24/134).

**Table 3:** Adverse drug reactions with an incidence ≥10%, dermatological toxicity, interstitial lung disease, and embolic and thrombotic events by dosage

Adverse drug reactions n (%)	4 mg/day QD				8 mg/day QD				8 mg/day BID				10 mg/day QD				12 mg/day QD				12 mg/day BID				16 mg/day QD				Total			
	*N* = 29				*N* = 38				*N* = 38				*N* = 18				*N* = 3				*N* = 5				*N* = 3				*N* = 134			
	Any grade		≥G3		Any grade		≥G3		Any grade		≥G3		Any grade		≥G3		Any grade		≥G3		Any grade		≥G3		Any grade		≥G3		Any grade		≥G3	
Platelet count decreased	13	(44.8)	2	(6.9)	29	(76.3)	4	(10.5)	26	(68.4)	4	(10.5)	12	(66.7)	2	(11.1)	2	(66.7)	1	(33.3)	5	(100)	3	(60)	3	(100)	2	(66.7)	90	(67.2)	18	(13.4)
Pyrexia	7	(24.1)	0		21	(55.3)	1	(2.6)	15	(39.5)	0		8	(44.4)	0		3	(100)	0		2	(40)	0		3	(100)	0		59	(44)	1	(0.7)
Rash	11	(37.9)	0		20	(52.6)	3	(7.9)	11	(28.9)	1	(2.6)	7	(38.9)	1	(5.6)	0		0		2	(40)	0		0		0		51	(38.1)	5	(3.7)
Diarrhea	6	(20.7)	0		9	(23.7)	1	(2.6)	10	(26.3)	1	(2.6)	9	(50)	0		3	(100)	0		1	(20)	1	(20)	2	(66.7)	1	(33.3)	40	(29.9)	4	(3)
Stomatitis	7	(24.1)	0		9	(23.7)	0		11	(28.9)	0		4	(22.2)	0		1	(33.3)	0		2	(40)	0		1	(33.3)	0		35	(26.1)	0	
Urticaria	5	(17.2)	0		5	(13.2)	0		8	(21.1)	1	(2.6)	6	(33.3)	1	(5.6)	2	(66.7)	1	(33.3)	3	(60)	0		1	(33.3)	1	(33.3)	30	(22.4)	4	(3)
Pruritus	8	(27.6)	0		11	(28.9)	0		5	(13.2)	0		4	(22.2)	0		0		0		0		0		1	(33.3)	0		29	(21.6)	0	
Hemoglobin decreased	4	(13.8)	0		9	(23.7)	1	(2.6)	7	(18.4)	1	(2.6)	4	(22.2)	1	(5.6)	0		0		3	(60)	1	(20)	1	(33.3)	0		28	(20.9)	4	(3)
Nausea	1	(3.4)	0		5	(13.2)	0		7	(18.4)	0		7	(38.9)	0		2	(66.7)	0		3	(60)	0		1	(33.3)	0		26	(19.4)	0	
Rash maculo-papular	4	(13.8)	1	(3.4)	11	(28.9)	1	(2.6)	8	(21.1)	1	(2.6)	0		0		1	(33.3)	0		1	(20)	0		1	(33.3)	0		26	(19.4)	3	(2.2)
ALT increased	6	(20.7)	0		8	(21.1)	0		5	(13.2)	1	(2.6)	5	(27.8)	1	(5.6)	1	(33.3)	0		0		0		0		0		25	(18.7)	2	(1.5)
Vomiting	3	(10.3)	0		3	(7.9)	0		4	(10.5)	1	(2.6)	7	(38.9)	1	(5.6)	2	(66.7)	0		3	(60)	0		2	(66.7)	0		24	(17.9)	2	(1.5)
AST increased	5	(17.2)	0		9	(23.7)	0		5	(13.2)	0		2	(11.1)	0		1	(33.3)	0		0		0		0		0		22	(16.4)	0	
Decreased appetite	0		0		4	(10.5)	0		5	(13.2)	0		6	(33.3)	0		1	(33.3)	0		2	(40)	0		1	(33.3)	0		19	(14.2)	0	
Malaise	3	(10.3)	0		4	(10.5)	0		5	(13.2)	0		3	(16.7)	0		1	(33.3)	0		1	(20)	0		0		0		17	(12.7)	0	
WBC decreased	2	(6.9)	0		5	(13.2)	0		8	(21.1)	0		0		0		0		0		0		0		2	(66.7)	0		17	(12.7)	0	
Hypoalbuminemia	4	(13.8)	0		5	(13.2)	0		4	(10.5)	0		2	(11.1)	0		0		0		1	(20)	0		0		0		16	(11.9)	0	
Dry skin	4	(13.8)	0		2	(5.3)	0		5	(13.2)	0		2	(11.1)	0		1	(33.3)	0		0		0		0		0		14	(10.4)	0	
Dermatological toxicity^a^	24	(82.8)	1	(3.4)	36	(94.7)	4	(10.5)	34	(89.5)	4	(10.5)	15	(83.3)	2	(11.1)	3	(100)	1	(33.3)	5	(100)	0		3	(100)	1	(33.3)	120	(89.6)	13	(9.7)
Interstitial lung disease^b^	1	(3.4)	1	(3.4)	2	(5.3)	0		4	(10.5)	2	(5.3)	0		0		0		0		2	(40)	2	(40)	1	(33.3)	1	(33.3)	10	(7.5)	6	(4.5)
Embolic and thrombotic events^c^	3	(10.3)	2	(6.9)	6	(15.8)	1	(2.6)	7	(18.4)	5	(13.2)	5	(27.8)	5	(27.8)	2	(66.7)	1	(33.3)	0		0		1	(33.3)	1	(33.3)	24	(17.9)	15	(11.2)
Venous thromboembolism	2	(6.9)	1	(3.4)	4	(10.5)	1	(2.6)	6	(15.8)	5	(13.2)	3	(16.7)	3	(16.7)	1	(33.3)	1	(33.3)	0		0		1	(33.3)	1	(33.3)	17	(12.7)	12	(9)
Pulmonary embolism	2	(6.8)	1	(3.4)	1	(2.6)	1	(2.6)	4	(10.5)	4	(10.5)	3	(16.7)	3	(16.7)	1	(33.3)	1	(33.3)	0		0		1	(33.3)	1	(33.3)	12	(8.9)	11	(8.2)
Other embolic and thrombotic events	1	(3.4)	1	(3.4)	2	(5.3)	0		1	(2.6)	0		2	(11.1)	2	(11.1)	2	(66.7)	0		0		0		0		0		8	(6)	3	(2.2)

*ALT* alanine aminotransferase, *AST* aspartate aminotransferase, *BID* twice daily, *QD* once daily, *WBC* white blood cell count

^a^Dermatological toxicity: Events classified as dermatological and subcutaneous tissue disorder in MedDRA (System Organ Class)

^b^Interstitial lung disease: includes lung disorder and pneumonitis

^c^Embolic and thrombotic events: Events classified as embolism and thrombosis in MedDRA (Standardised MedDRA Queries)

The proportion of patients whose treatment was interrupted was 44.7% (17/38) in the 8 mg/day BID (MTD) group and 66.7% (12/18) in the 10 mg/day QD (MTD) group. The proportion of patients whose dose was decreased was 18.4% (7/38) in the 8 mg/day BID (MTD) group and 38.9% (7/18) in the 10 mg/day QD (MTD) group. The incidence of discontinuation due to ADRs at the MTD level was 11.1% (2/18) with TAS-121 10 mg/day QD and 7.9% (3/38) with TAS-121 8 mg/day BID. No treatment-related deaths occurred during the study.

### Pharmacokinetics

The PK parameters are shown in Table 4. The plasma concentration-time profile of TAS-121 is shown in Supplementary Fig. 2 (Online Resource 4).

**Table 4:** Pharmacokinetic parameters (cycle 1, day 15)

Regimen	Dose	n	Mean (standard deviation)				
			T_1/2_ (h)	T_max_^a^ (h)	C_max_ (ng/mL)	AUC_0-12h_ (ng•h/mL)	AUC_0-24h_ (ng•h/mL)
QD	4 mg/day	3	8.83 (2.88)	1.00 (1.00–1.00)	304 (13)	NA	2670 (1110)
	8 mg/day	9	8.60 (2.49)	1.00 (0.50–2.00)	569 (312)	NA	4860 (2520)
	10 mg/day	7	8.61 (1.73)	2.00 (0.75–4.00)	900 (376)	NA	8040 (4190)
	12 mg/day	2	8.12 (NA)^b^	1.12 (1.00–1.23)	1180 (NA)	NA	9500 (NA)
	16 mg/day	1	6.44 (NA)	2.00 (NA)	825 (NA)	NA	5870 (NA)
BID	8 mg/day	9	5.11 (1.48)	1.00 (0.50–1.00)	290 (104)	1520 (520)	NA
	12 mg/day	4	8.84 (3.31)	2.00 (1.00–3.00)	772 (201)	5860 (1460)	NA

*AUC*_*0-12h*_ area under the plasma concentration time curve from time 0 to 12 h, *AUC*_*0-24h*_ area under the plasma concentration time curve from time 0 to 24 h, *BID* twice daily, *C*_*max*_ maximum plasma concentration, *NA* not applicable, *QD* once daily, *T*_*1/2*_ elimination half-life, *T*_*max*_ time to maximum plasma concentration

^a^median (minimum–maximum)

^b^The T_1/2_ of 12 mg/day was based on one patient. The absolute value of the correlation coefficient was less than 0.9 in another patient, so the T_1/2_ in the patient was not calculated

### Efficacy

**Fig. 2:**
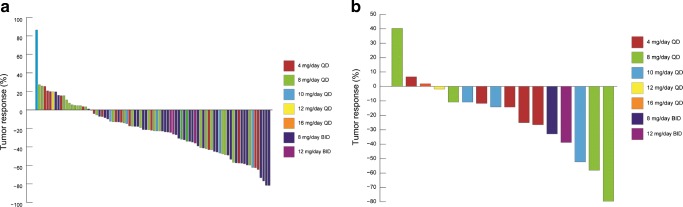
**Tumor response in T790M-positive (a) and T790M-negative (b) patients**. *Abbreviations: BID* twice daily, *QD* once daily

The central review results of tumor response in T790M-positive and T790M-negative patients are shown in Fig. 2a and b, respectively, and the results of ORR and DCR in T790M-positive and T790M-negative patients are shown in Supplementary Table 2 (Online Resource 5). Among T790M-positive patients (patients with measurable lesions, *n* = 86), the ORR for all patients (regardless of the dosage) was 28%, and was highest at 8 mg/day BID (39%). Among T790M-negative patients (*n* = 16), the ORR for all patients was 19%.

The Kaplan–Meier curve of PFS in T790M-positive patients is shown in Supplementary Fig. 3 (Online Resource 6). The median PFS was 165, 125, 253, and 401 days in the 4 mg/day QD, 8 mg/day QD, 8 mg/day BID, and 10 mg/day QD groups, respectively. Of note, the number of patients and events in the 10 mg/day QD group (seven patients/three events) was notably lower compared with those in the other groups (23 patients/17 events, 33 patients/25 events, and 36 patients/21 events in the 4 mg/day QD, 8 mg/day QD, and 8 mg/day BID groups, respectively).

In Cohort C (seven patients who had previously received osimertinib treatment), QD dosing was used because the incidence of interstitial lung disease and Grade 3 embolic and thrombotic events was lower with QD dosing versus BID dosing. Cohort C started with 8 mg/day QD and one patient was registered. Subsequently, the tolerability of 10 mg/day QD was confirmed, and six patients were registered to the 10 mg/day QD group. Of the seven patients in Cohort C, the best responses were stable disease in two patients, PD in three patients, and two patients were not evaluable.

## Discussion

This is the first-in-human phase I study to evaluate the safety and tolerability, PK, and efficacy of TAS-121. The tolerability of TAS-121 10 mg/day QD and 8 mg/day BID was confirmed. The incidence of discontinuation due to ADRs at the MTD levels with both QD and BID was low and a good safety profile was shown.

As the frequency of ADRs and that of higher grade ADRs tended to be higher with dosage increase (4, 8, and 16 mg/day QD), BID dosing was also investigated for alleviating toxicity. The incidence of interstitial lung disease and Grade 3 embolic and thrombotic events tended to be higher with 8 mg/day BID dosing than with 8 mg/day QD dosing (interstitial lung disease: 10.5% versus 5.2%; Grade 3 embolic and thrombotic events: 13.2% versus 2.6%), although the incidence of pyrexia was lower with 8 mg/day BID dosing than with 8 mg/day QD dosing (39.5% versus 55.3%). Therefore, we cannot conclude that the safety profile of TAS-121 was superior with BID versus QD dosing.

In the present study, ADRs that were different from those previously reported for EGFR-TKIs were found, such as allergy-like dermatological toxicity, pyrexia, platelet count decreased, and embolic and thrombotic events. The most frequently reported AEs with currently approved EGFR-TKIs (gefitinib, erlotinib, afatinib, and osimertinib) include diarrhea, rash/acne, and dry skin [4, 6, 15, 16].

In the present study, dermatological toxicities and pyrexia tended to appear in the early period of TAS-121 administration. Furthermore, some patients presented both dermatological toxicity and pyrexia. Dermatological toxicity is known to be a major ADR related to EGFR-TKIs [17]. While allergy-like dermatological toxicities (e.g., urticaria) were observed in the present study, acneiform eruption has been reported with conventional EGFR-TKIs [18]. The allergy-like dermatological toxicities may have been caused by the off-target activity of TAS-121. In a nonclinical study, TAS-121 was found to affect the immune system, as induction of cytokines, including monocyte chemoattractant protein-1 (MCP-1), was confirmed in rats (Supplementary Table 3; Online Resource 7). MCP-1 levels are known to increase in allergic conditions [19], and this is thought to be associated with allergy-like dermatological toxicity.

Furthermore, increased inflammatory cytokine levels could contribute to an increased risk of thromboembolism by inducing intravascular inflammation. However, increased incidence of thromboembolism was not observed in the nonclinical study (unpublished observation). Nonclinical data cannot explain the incidence of platelet count decrease found in the present study; thus, this warrants further study.

The incidence of interstitial lung disease was 7.4% in the present study, but all patients recovered from this event, and no deaths occurred. In the present study, an interstitial lung disease assessment committee was set up to assess patients in whom interstitial lung disease was suspected. In seven patients, interstitial lung disease and pleural effusion or pulmonary congestion/edema were observed. These patients presented with pneumonopathy and pleural effusion, which differ from the pulmonary symptoms observed with conventional EGFR-TKIs (diffuse alveolar damage), and the possibility of TAS-121 causing pulmonary capillary leak-like symptoms was suggested. Patients in the present study recovered with steroids or study drug discontinuation.

The efficacy of TAS-121 in T790M-positive patients was evaluated in the present study. In some patients, tumor regression was confirmed in T790M-positive patients, but the response rate was lower than expected. A possible reason for this is that the MTD was determined by off-target toxicity unrelated to EGFR inhibition, and the efficacy of TAS-121 in inhibiting EGFR was not maximized. Among T790M-positive patients, the mutation was detected using cfDNA for 96.3% (103/107) because re-biopsy was not mandatory in this study. Considering the diagnostic systems currently in development, which will be available in the near future, in this study, we assessed T790M mutation status using plasma samples (rather than biopsy) from the beginning. Advantages of liquid biopsy are that the burden on the patient is small and the possibility that T790M mutations can be detected even when the tissue itself cannot be collected or the tumor is uneven. A previous study has demonstrated that patients with T790M mutations have longer survival with osimertinib, regardless of whether the mutations are detected in plasma or in tissue [20], supporting the use of this approach.

The PK of TAS-121 was evaluated and the AUC of TAS-121, even at the lowest dose, was notably higher than that of the effective dose in the preclinical tumor xenograft model (906 ng•h/mL) (unpublished data).

Cohort C was included in the extension phase to evaluate the efficacy of TAS-121 in patients whose disease had previously progressed during osimertinib treatment, because PR was observed among patients in the dose escalation phase previously treated with osimertinib and osimertinib showed the efficacy after the treatment with rociletinib [21]. However, no clear evidence of efficacy was observed among the seven registered patients.

The results of the present study should be interpreted with consideration of the study limitations. The present study was limited by the open-label, non-randomized design and lack of an active comparator. The generalizability of our findings to other ethnic populations is limited.

In conclusion, TAS-121 was well tolerated up to the MTD and demonstrated antitumor activity in Japanese T790M-positive NSCLC patients.

## Electronic supplementary material

ESM 1 (DOCX 98 kb)

ESM 2 (DOCX 21 kb)

ESM 3 (DOCX 48 kb)

ESM 4 (DOCX 82 kb)

ESM 5 (DOCX 50 kb)

ESM 6 (DOCX 70 kb)

ESM 7 (DOCX 48 kb)
