# Supplementary material for: Phase I study of TAS-121, a third-generation epidermal growth factor receptor (EGFR) tyrosine kinase inhibitor, in patients with non-small-cell lung cancer harboring EGFR mutations
Source: Invest New Drugs. 2019 Feb 21;37(6):1207–17. doi: 10.1007/s10637-019-00732-4 (PMC6856039; doi:10.1007/s10637-019-00732-4)

**Online Resource 1**

**Supplementary Fig. 1. Study design**

Abbreviations: EGFR, epidermal growth factor receptor; MTD, maximum tolerated dose; PK, pharmacokinetics


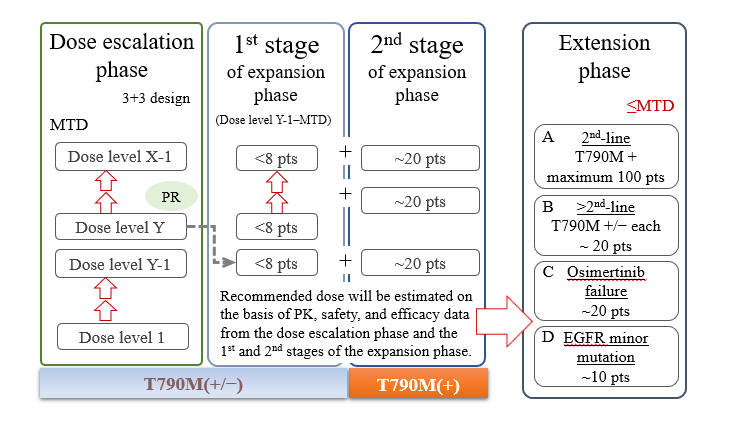

Supplement: Supplementary file 1 — (DOCX 98 kb) [file 10637_2019_732_MOESM1_ESM.docx]
