# Supplementary material for: Phase I study of TAS-121, a third-generation epidermal growth factor receptor (EGFR) tyrosine kinase inhibitor, in patients with non-small-cell lung cancer harboring EGFR mutations
Source: Invest New Drugs. 2019 Feb 21;37(6):1207–17. doi: 10.1007/s10637-019-00732-4 (PMC6856039; doi:10.1007/s10637-019-00732-4)
Supplement: Supplementary file 2 — (DOCX 21 kb) [file 10637_2019_732_MOESM2_ESM.docx]

**Online Resource 2**

**Supplementary methods. Complete list of inclusion and exclusion criteria**

**Inclusion Criteria**

***Inclusion criteria applicable to all phases***

A patient must meet all of the following inclusion criteria to be eligible for enrollment in this study:

Investigator or Sub-Investigator should observe the conditions of patients under hospitalization or equivalent to hospitalization from Day 1 through Day 21 of Cycle 1.

1. Provided written informed consent for treatment.

2. Male or female ≥20 years at enrollment.

3. Histologically or cytologically confirmed NSCLC

4. Eastern Cooperative Oncology Group performance status of 0 or 1 at time of patient’s allocation.

5. Able to take medications orally (e.g., no feeding tube).

6. Adequate organ function as defined by the following criteria:

a) AST and ALT ≤3 × upper limit of normal (ULN)

b) Total serum bilirubin of ≤1.5 × ULN

c) ANC ≥1 500/mm3 (i.e., ≥1.5 × 109/L by the International System of Units [SI])

(excluding measurements obtained within 7 days after administration of granulocyte colony-stimulating factor)

d) Platelet count ≥75 000/mm3 (SI unit: ≥75 × 109/L) (excluding measurements obtained within 7 days after a transfusion of platelets)

e) Hemoglobin value of ≥8.0 g/dL, excluding measurements within 4 weeks after a transfusion of packed red blood cells or whole blood

f) Blood oxygen saturation level ≥90%

g) Creatinine clearance (CrCl) of ≥50 mL/min

Calculate CrCl from serum creatinine (Cockcroft-Gault Equation):

$$CrCl=\frac{\left( 140 - age \right)x \left( body weight\left[ kg \right] \right)}{(72 x serum creatinine[mg/dL])} x (0.85[female] or 1.00[male])$$

7. A life expectancy of at least 60 days

***Inclusion criteria for dose escalation phase and 1st stage of expansion phase***

1. Have documented evidence of any activating mutation (G719X, deletion in exon 19, L858R, L861Q) in the EGFR.

2. Have had previous treatment with a single-agent EGFR TKI (e.g., gefitinib, erlotinib, neratinib, dacomitinib, or afatinib).

3. Without standard treatment remains

***Inclusion criteria for 2nd stage of expansion phase***

1. Have documented evidence of any activating mutation (G719X, deletion in exon 19, L858R, or L861Q) in the EGFR.

2. Have had previous treatment with a single-agent EGFR TKI (e.g., gefitinib, erlotinib, neratinib, dacomitinib, or afatinib).

3. At least one measurable lesion based on Response Evaluation Criteria in Solid Tumors (RECIST) Version 1.18.

4. With T790M mutation in EGFR as determined by PCR-based testing of either a blood or tumor sample, using the Sponsor’s central laboratory in the baseline period.

***Inclusion criteria for extension phase Cohort A***

1. Have documented evidence of any activating mutation (except for exon 20 insertion mutation) in the EGFR.

2. At least one measurable lesion based on RECIST (Version 1.18).

3. Have had previous treatment with a single-agent EGFR TKI (gefitinib, erlotinib, or afatinib) as first line EGFR TKI treatment without any further treatment.

4. Radiological documentation of disease progression following the first line EGFR TKI treatment with NSCLC, and allocation within 30 days after the EGFR-TKI discontinuation.

5. With T790M mutation in EGFR as determined by PCR-based testing of a blood sample using the Sponsor’s central laboratory in the baseline period.

***Inclusion criteria for extension phase Cohort B***

1. Have documented evidence of any activating mutation (except for exon 20 insertion mutation) in the EGFR.

2. At least one measurable lesion based on RECIST (Version 1.18).

3. Have had two or more prior therapies for NSCLC and treated with gefitnib, erlotinib, or afatinib immediately before allocation.

4. Radiological documentation of disease progression following immediate prior EGFR TKI treatment with NSCLC, and allocation within 30 days after the EGFR-TKI discontinuation.

***Inclusion criteria for extension phase Cohort C***

1. Have documented evidence of any activating mutation (except for exon 20 insertion mutation) in the EGFR.

2. At least one measurable lesion based on RECIST (Version 1.18).

3. Have had osimertinib immediately before allocation and radiological documentation of disease progression.

***Inclusion criteria for extension phase Cohort D***

1. Have documented evidence of G719X activating mutation in the EGFR.

2. At least one measurable lesion based on RECIST (Version 1.18).

Note: In the Extension Phase, a patient who has confirmed G719X activating mutation in the EGFR should be allocated to Cohort A preferentially, followed in order by Cohort B and Cohort D.

**7.2 Exclusion Criteria**

A patient should be excluded from this study if any of the following conditions are observed:

1. Treatment with EGFR TKIs within the following time frame prior to the day on which the study drug is scheduled to be administered:

a) reversible EGFR TKIs (gefitinib or erlotinib) within 5 days

b) irreversible EGFR TKIs (neratinib, dacomitinib, or afatinib) within 14 days

c) irreversible EGFR TKIs (osimertinib) within 14 days (only applies to Cohort C of the Extension Phase)

2. Prior treatment with an EGFR-T790M inhibitor (e.g. osimertinib, rociletinib, ASP8273) (applies to the 2nd stage of the Expansion Phase and the Cohort A, B and D of the Extension Phase).

3. Current evidence of corneal disorder/keratopathy, including but not limited to bullous/band keratopathy, corneal abrasion, inflammation/ulceration, and keratoconjunctivitis, confirmed by ophthalmological examination.

4. History or current evidence of cardiac arrhythmia and/or conduction abnormality.

5. Treatment with any of the following within the specified time frame prior to the day on which the study drug is scheduled to be administered:

a) Major surgery within 4 weeks prior to scheduled treatment (the surgical incision should be fully healed prior to the day on which the study drug is scheduled to be administered).

b) Extended-field radiotherapy within 4 weeks or limited-field radiotherapy within 2 weeks of scheduled treatment.

c) Any anticancer treatment except for EGFR TKIs (gefitinib or erlotinib) within

2 weeks of scheduled treatment (mitomycin within 5 weeks prior to scheduled treatment.)

d) Any investigational agent received either concurrently or within the last 2 weeks

6. Unresolved toxicity of ≥Grade 1 attributed to any prior therapies (excluding alopecia and skin pigmentation).

7. Vomiting within 24 hours prior to the day on which study drug is scheduled to be administered.

8. A serious illness or medical condition(s) including, but not limited to, the following:

a) Known brain metastasis that is not stable and with clinical symptoms or requiring treatment

b) Known leptomeningeal metastasis

c) Known acute systemic infection

d) Known medical history of interstitial lung disease/drug-induced interstitial lung disease/radiation pneumonitis which required steroid treatment/any evidence of clinically active interstitial lung disease

e) Myocardial infarction, severe/unstable angina, symptomatic congestive heart failure (New York Heart Association class III or IV) within the previous 6 months; if >6 months, then cardiac function must be within normal limits and the patient must be free of cardiac-related symptoms

f) Chronic nausea, vomiting, or diarrhea considered to be clinically significant in the opinion of the Investigator or Sub-investigator

g) Known severe chronic kidney disease

h) Known positivity of human immunity deficiency (HIV) antibody, hepatitis B virus surface antigen, or hepatitis C virus (HCV) antibody in baseline virus test. In addition, a patient who is known to be HCV RNA negative is eligible, even if HCV antibody positive.

i) Other severe acute or chronic medical or psychiatric condition or laboratory abnormality that may increase the risk associated with study participation or study drug administration, or may interfere with the interpretation of the study results, based on the judgment of the Investigator or Sub-investigator who would determine if the patient is inappropriate for entry into this study

9. Known hypersensitivity to any drugs similar to TAS-121 in structure or class

10. Prior therapy with TAS-121 (except for patients enrolled in Level 1 of the Dose Escalation Phase)

11. Previous or concurrent cancer that is distinct in primary disease or histology from the cancer being evaluated in this study, except cervical carcinoma in situ, treated basal cell carcinoma, superficial bladder tumors (stage Ta, Tis and T1), cancers corresponding to intraepithelial or intramucosal neoplasia, or any cancer curatively treated >5 years prior to the day on which the study drug is scheduled to be administered.

12. Pregnant or lactating female (including the cessation of lactation) or women of

child-bearing potential who have a positive pregnancy test (urine or serum) within

7 days prior to the day on which study drug is scheduled to be administered. Males and females who do not agree to use adequate birth control if conception is possible during the clinical study and for 180 days after the last dose. Female patients are considered not to be of child-bearing potential if they have a history of tubal ligation or hysterectomy or are post-menopausal with a minimum of 1 year without menses (without usage of medications due to medical justifications).

13. Requirement for chronic oxygen therapy for chronic obstructive pulmonary disease or pleural effusions (malignant or benign).

14. Uncontrollable pleural effusion

15. Prolonged QTc interval on pre-entry ECG >450 msec.

16. Three or more prior EGFR TKI treatments (regimens or agents)(only applies to the Cohort B of the Extension Phase).

17. Three or more prior therapies with NSCLC (only applies to the Cohort D of the Extension Phase)

18. Neo-adjuvant or adjuvant therapy within 6 months prior of starting 1st EGFR TKI treatment (only applies to Cohort A of the Extension Phase)

19. History or current evidence of symptoms of obvious venous thrombosis

20. Current evidence of pulmonary artery thrombosis by imaging

**7.3 Replacement Criteria**

If the patient does not meet the definition of the DLT Evaluable Patients in Table 17.2.1-1, they will be replaced following agreement between the Investigator and the Sponsor. Patients will not be replaced in the Expansion Phase.
