# Supplementary material for: Phase I study of TAS-121, a third-generation epidermal growth factor receptor (EGFR) tyrosine kinase inhibitor, in patients with non-small-cell lung cancer harboring EGFR mutations
Source: Invest New Drugs. 2019 Feb 21;37(6):1207–17. doi: 10.1007/s10637-019-00732-4 (PMC6856039; doi:10.1007/s10637-019-00732-4)
Supplement: Supplementary file 3 — (DOCX 48 kb) [file 10637_2019_732_MOESM3_ESM.docx]

**Online Resource 3**

**Supplementary Table 1. Main analysis sets in each phase**

| **Analysis Set** | **Definition** |
| --- | --- |
| **Dose escalation phase** | |
| All enrolled patients | All patients enrolled in the dose escalation phase. |
| All treated patients | All patients who received at least one dose of TAS-121. |
| DLT-evaluable patients | Patients who received the study treatment in the dose escalation phase excluding those who meet any of the following criteria:  1. Being found to have not met the inclusion criteria applicable to all phases or inclusion criteria for the dose escalation phase and first stage of the expansion phase after enrollment.  2. Of patients who did not show DLT, those for whom the duration of the study treatment was <17 days in Cycle 1 on QD dosing, the number of the study dose was <34 doses in Cycle 1 on BID dosing for reasons other than therapeutic actions for AEs.  3. No specified text or observation was implemented in Cycle 1.  4. Prohibited concomitant medications or therapies were administered in Cycle 1. |
| **First stage of the expansion phase** | |
| All enrolled patients | All patients enrolled in the dose escalation phase and the first stage of the expansion phase. |
| All treated patients | All patients who received at least one dose of TAS-121. |
| Full analysis set | Of the patients who received study treatment, all patients who fulfilled the inclusion criteria applicable to all phases, or the inclusion criteria for the dose escalation phase and first stage of the expansion phase, and who were evaluated for at least one efficacy endpoint after administration of the study drug. |
| **Second stage of the expansion phase** | |
| All enrolled patients | All patients enrolled in the second stage of the expansion phase. |
| All treated patients | All patients who received at least one dose of TAS-121. |
| Full analysis set | Of the patients who received study treatment in the second stage of the expansion phase, all patients who fulfilled the inclusion criteria applicable to all phases, and the inclusion criteria for the second stage of the expansion phase, and who were evaluated for at least one efficacy endpoint after administration of the study drug. |
| **Extension phase** | |
| All enrolled patients | All patients enrolled in the extension phase. |
| All treated patients | All patients who received at least one dose of TAS-121. |
| Full analysis set | Of the patients who received study treatment in the extension phase, all patients who fulfilled the following inclusion criteria and who were evaluated for at least one efficacy endpoint after administration of the study drug:  Cohort A: inclusion criteria applicable to all phases and inclusion criteria for Cohort A of the extension phase  Cohort B: inclusion criteria applicable to all phases and inclusion criteria for Cohort B of the extension phase  Cohort C: inclusion criteria applicable to all phases and inclusion criteria for Cohort C of the extension phase  Cohort D: inclusion criteria applicable to all phases and inclusion criteria for Cohort D of the extension phase |
