# Supplementary material for: Phase I study of TAS-121, a third-generation epidermal growth factor receptor (EGFR) tyrosine kinase inhibitor, in patients with non-small-cell lung cancer harboring EGFR mutations
Source: Invest New Drugs. 2019 Feb 21;37(6):1207–17. doi: 10.1007/s10637-019-00732-4 (PMC6856039; doi:10.1007/s10637-019-00732-4)

**Online Resource 4**

**Supplementary Fig. 2. Plasma concentration-time profile of TAS-121 (Cycle 1, Day 15)**

^a^ In the 8 mg/day QD group, n = 8 at the 1-h timepoint only.

^b^ In the 10 mg/day QD group, n = 6 at the 0.5-h timepoint only.

^c^ In the 12 mg/day QD group n = 1 at the 1-h timepoint only.

Abbreviations: QD, once daily; BID, twice daily


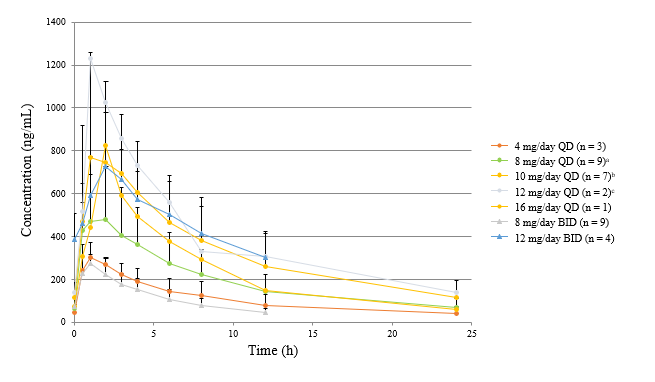

Supplement: Supplementary file 4 — (DOCX 82 kb) [file 10637_2019_732_MOESM4_ESM.docx]
