# Supplementary material for: Phase I study of TAS-121, a third-generation epidermal growth factor receptor (EGFR) tyrosine kinase inhibitor, in patients with non-small-cell lung cancer harboring EGFR mutations
Source: Invest New Drugs. 2019 Feb 21;37(6):1207–17. doi: 10.1007/s10637-019-00732-4 (PMC6856039; doi:10.1007/s10637-019-00732-4)
Supplement: Supplementary file 5 — (DOCX 50 kb) [file 10637_2019_732_MOESM5_ESM.docx]

**Online Resource 5**

**Supplementary Table 2. Response rates in T790M-positive and T790M-negative patients**

| Response, n (%) | 4  mg/day QD | 8  mg/day QD | 8  mg/day BID | 10 mg/day QD | 12 mg/day QD | 12 mg/day BID | 16 mg/day QD | Total |
| --- | --- | --- | --- | --- | --- | --- | --- | --- |
| T790M-positive | N = 15 | N = 28 | N = 33 | N = 7 | N = 1 | N = 3 | N = 0 | N = 87 |
| Best overall response rate |  |  |  |  |  |  |  |  |
| Partial response | 4 (26.7) | 6 (21.4) | 13 (39.4) | 1 (14.3) | 0 (0.0) | 0 (0.0) | - | 24 (27.6) |
| Stable disease | 3 (20.0) | 11 (39.3) | 11 (33.3) | 3 (42.9) | 0 (0.0) | 1 (33.3) | - | 29 (33.3) |
| Progression disease | 7 (46.7) | 11 (39.3) | 8 (24.2) | 3 (42.9) | 1 (100.0) | 1 (33.3) | - | 31 (35.6) |
| Not evaluable | 1 (6.7) | 0 (0.0) | 1 (3.0) | 0 (0.0) | 0 (0.0) | 1 (33.3) | - | 3 (3.4) |
| Objective response rate | 4 (26.7) | 6 (21.4) | 13 (39.4) | 1 (14.3) | 0 (0.0) | 0 (0.0) | - | 24 (27.6) |
| Disease control rate | 7 (46.7) | 17 (60.7) | 24 (72.7) | 4 (57.1) | 0 (0.0) | 1 (33.3) | - | 53 (60.9) |
| T790M-negative | N = 5 | N = 4 | N = 1 | N = 3 | N = 1 | N = 1 | N = 1 | N = 16 |
| Best overall response rate |  |  |  |  |  |  |  |  |
| Partial response | 0 (0.0) | 2 (50.0) | 0 (0.0) | 1 (33.3) | 0 (0.0) | 0 (0.0) | 0 (0.0) | 3 (18.8) |
| Stable disease | 2 (40.0) | 1 (25.0) | 1 (100.0) | 2 (66.7) | 0 (0.0) | 1 (100.0) | 0 (0.0) | 7 (43.8) |
| Progression disease | 2 (40.0) | 1 (25.0) | 0 (0.0) | 0 (0.0) | 0 (0.0) | 0 (0.0) | 1 (100.0) | 4 (25.0) |
| Not evaluable | 1 (20.0) | 0 (0.0) | 0 (0.0) | 0 (0.0) | 1 (100.0) | 0 (0.0) | 0 (0.0) | 2 (12.5) |
| Objective response rate | 0 (0.0) | 2 (50.0) | 0 (0.0) | 1 (33.3) | 0 (0.0) | 0 (0.0) | 0 (0.0) | 3 (18.8) |
| Disease control rate | 2 (40.0) | 3 (75.0) | 1 (100.0) | 3 (100.0) | 0 (0.0) | 1 (100.0) | 0 (0.0) | 10 (62.5) |
