# Supplementary material for: Phase I study of TAS-121, a third-generation epidermal growth factor receptor (EGFR) tyrosine kinase inhibitor, in patients with non-small-cell lung cancer harboring EGFR mutations
Source: Invest New Drugs. 2019 Feb 21;37(6):1207–17. doi: 10.1007/s10637-019-00732-4 (PMC6856039; doi:10.1007/s10637-019-00732-4)

**Online Resource 6**

**Supplementary Fig. 3. Kaplan–Meier curve of progression-free survival in T790M-positive patients with doses lower than or equal to the maximum tolerated dose**

Abbreviations: PFS, progression-free survival; QD, once daily; BID, twice daily


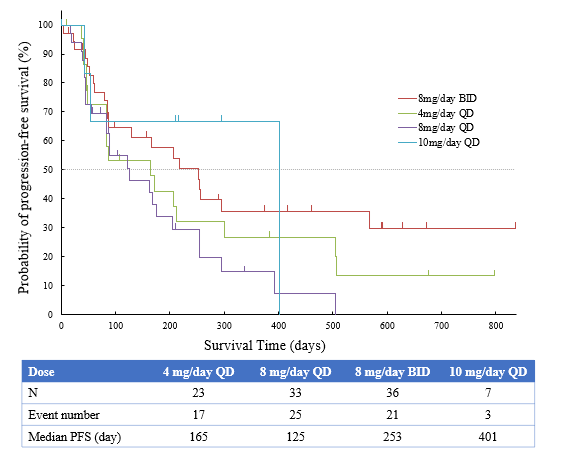

Supplement: Supplementary file 6 — (DOCX 70 kb) [file 10637_2019_732_MOESM6_ESM.docx]
