# Supplementary material for: Phase I study of TAS-121, a third-generation epidermal growth factor receptor (EGFR) tyrosine kinase inhibitor, in patients with non-small-cell lung cancer harboring EGFR mutations
Source: Invest New Drugs. 2019 Feb 21;37(6):1207–17. doi: 10.1007/s10637-019-00732-4 (PMC6856039; doi:10.1007/s10637-019-00732-4)
Supplement: Supplementary file 7 — (DOCX 48 kb) [file 10637_2019_732_MOESM7_ESM.docx]

**Online Resource 7**

**Supplementary Table 3.** Effect of TAS-121 in the immune system

|  | Non-treatment group | Vehicle group | Testing group |
| --- | --- | --- | --- |
| Number of serum samples | 3 | 3 | 3 |
| IL-1α | 134.54 (24.41) | 142.03 (70.55) | 110.05 (12.12) |
| MCP-1 | 377.32 (36.65) | 324.61 (43.48) | 557.92 (50.51)** |
| TNF-α | 42.38 (2.73) | 35.51 (16.59) | 92.07 (33.36) |
| IFN-α | 13.47 (1.74) | 12.04 (4.45) | 19.09 (6.01) |
| IL-4 | 0.02 (0.03) | 0.02 (0.03) | 2.38 (2.10) |

Data are presented as mean (standard deviation) [pg/mL].

** p ≤0.01 versus vehicle-administered group

Abbreviations: IL, interleukin; MCP-1, monocyte chemoattractant protein-1, TNF-α, tumor necrosis factor-α; IFN-α, interferon-α
